# Beyond the Usual Suspects: A Challenging Case of Disseminated Sporotrichosis Masquerading as Suppurative Lymphadenitis in a Dialysis‐Dependent Chronic Kidney Disease Patient With Lupus

**DOI:** 10.1155/crdi/2428389

**Published:** 2026-10-07

**Authors:** Natalia Chilinque Zambão da Silva, Jordanna de Paula Mendes Torres, Valéria Regina Soares, Nathalia Antônio de Oliveira Velasco, Violeta Duarte Tortelly Costa, Bruno Carrilho Hollanda

**Affiliations:** ^1^ Department of Clinical Medicine Fluminense Federal University Niterói Rio de Janeiro Brazil uff.br; ^2^ Department of Clinical Medicine Niterói Hospital Complex Niterói Rio de Janeiro Brazil

**Keywords:** immunocompromised host, sporotrichosis, systemic lupus erythematosus

## Abstract

Sporotrichosis, a subcutaneous mycosis, can manifest as severe disseminated disease in immunocompromised hosts. This report describes a challenging case of extracutaneous sporotrichosis in a 28‐year‐old female with systemic lupus erythematosus (SLE) on immunosuppressive therapy, who also faced significant socioeconomic constraints. The patient initially presented to the emergency department with a painful right inguinal mass. Physical examination revealed a hemorrhagic lesion, and aspiration of the material was performed for culture, which returned negative. She was discharged but returned 2 months later with worsening symptoms and widespread lesions. During this second admission, a detailed history finally revealed contact with cats. The patient was readmitted for an inguinal lymph node biopsy. Histopathological analysis revealed granulomatous lymphadenitis with stellate necrosis, and the diagnosis was ultimately confirmed by culture, identifying *Sporothrix schenckii* complex. Treatment with itraconazole was initiated but later switched to liposomal amphotericin B due to hepatotoxicity. The patient completed a 1‐year course of liposomal amphotericin B with an excellent clinical response. This case highlights the diagnostic complexity of sporotrichosis in patients with SLE, its potential for severe dissemination, and the impact of socioeconomic barriers on timely diagnosis and disease management.

## 1. Introduction

Sporotrichosis, the most common subcutaneous mycosis in Latin America [1], is also considered a neglected disease. Its etiological agent belongs to the genus *Sporothrix spp.*, which includes 53 species, with *S. brasiliensis*, *S. schenckii*, and *S. globosa* being particularly relevant to public health [2]. In Brazil, the state of Rio de Janeiro stands out as the primary epicenter, as it was here that the first epidemic of cat‐transmitted sporotrichosis emerged in 1998 [3]. The more aggressive forms of sporotrichosis, such as disseminated cutaneous and extracutaneous, are observed more frequently in immunosuppressed patients [4]. In individuals with compromised immune systems, such as those with systemic lupus erythematosus (SLE), the inflammatory and phagocytic responses are less efficient, allowing the disease to spread more easily. The impaired production of proinflammatory cytokines, such as IFN‐γ and TNF‐α, hinders macrophage activation, facilitating the invasion of deep tissues and systemic organs [5]. We report an unusual form of extracutaneous sporotrichosis in an immunocompromised patient with SLE. She was successfully treated with liposomal amphotericin B.

## 2. Case Presentation

A 28‐year‐old woman patient with a history of SLE, Systemic Lupus Erythematosus Disease Activity Index (score 6), who is currently using mycophenolate and prednisone. She has had chronic kidney disease since age 14, with multiple episodes of vascular access failure and multiple tunneled catheter replacements. She also reports a prior diagnosis of endometriosis and anxiety‐depressive disorders. She had negative serology for hepatitis C, tested negative for HIV, and was vaccinated against hepatitis B with documented seroconversion.

The patient was admitted in April 2024 with a 1‐week history of a painful, progressively enlarging mass in the right inguinal region, associated with discomfort upon ambulation. On admission, she was afebrile and hemodynamically stable. The lesion is shown in Figures 1 and 2. A diagnostic puncture of the lesion was performed, which yielded a hemorrhagic collection. Cultures were promptly obtained from the aspirate. Empirical antibiotic therapy was initiated with vancomycin and amikacin, following the recommendation of the hospital infection control team, to provide broad‐spectrum coverage for potential Gram‐positive and Gram‐negative pathogens.

**FIGURE 1:**
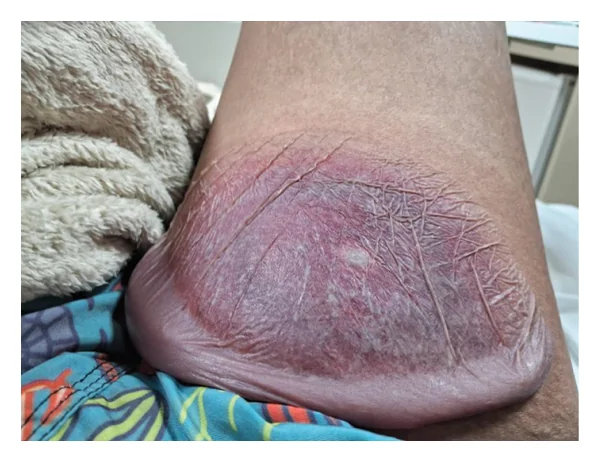
Anterior view photograph of the lesion located in the medial third of the thigh.

**FIGURE 2:**
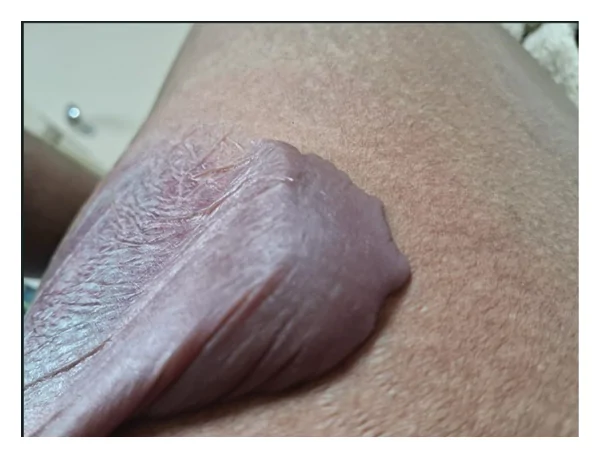
Lateral view photograph of the lesion located in the medial third of the thigh.

Following drainage, with final culture results returning negative and after completing 7 days of antimicrobial therapy, the patient remained hospitalized for adjustments related to her dialysis catheter. After 2 months of hospitalization, the patient presented again with pain and edema in the inguinal region. At this time, she reported to the medical team that she had 21 cats in her household and with the return of pain in the inguinal region, and she underwent an ultrasound for further evaluation.

Ultrasound performed in June 2024 revealed a lymph node in the right thigh measuring 4.1 × 2.3 × 1.3 cm, with surrounding perilesional edema (Figure 3). Abdominal computed tomography (CT) showed retroperitoneal lymphadenopathy and hypodense renal lesions with calcified foci, in addition to lytic lesions in the iliac bones and femoral heads. The lytic bone lesions were deemed nonspecific and attributable to the patient’s chronic kidney disease–related metabolic alterations, not to sporotrichosis, and were not further investigated. An ultrasound‐guided lymph node biopsy was performed by interventional radiology on June 11, 2024, without complications.

**FIGURE 3:**
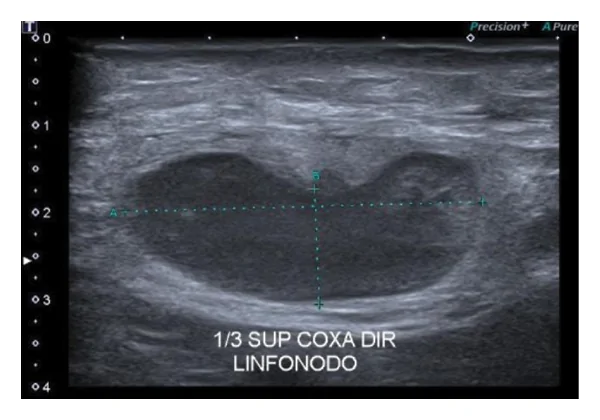
Lymph node ultrasound: dimension A: 41.1, B: 13.3 mm.

The patient was discharged 2 days postbiopsy to await the results in an outpatient setting. However, 3 days after discharge, the patient returned on June 16, 2024, due to worsening pain in the right thigh, along with systemic symptoms including unmeasured low‐grade evening fever, malaise, and loss of appetite. Physical examination revealed four nonpruritic, painless ulcerated skin lesions with well‐defined borders and clean bases on the right knee, some of which were in the healing phase; however, overall clinical deterioration was noted.

At this time, the histopathological results from the inguinal lymph node biopsy became available. The analysis revealed granulomatous lymphadenitis with stellate necrosis; however, special stains for acid‐fast bacilli (AFB) and fungi (PAS, Grocott) were negative. The diagnosis was ultimately confirmed by a subsequent culture, which identified the *Sporothrix schenckii* complex. Microbiological identification was performed by Matrix‐Assisted Laser Desorption/Ionization–Time of Flight (MALDI‐TOF) mass spectrometry. Antifungal therapy with itraconazole (200 mg twice daily) was promptly initiated. According to the sporotrichosis infection classification, the case was defined as disseminated cutaneous sporotrichosis.

On August 11, 2024, the patient developed clinical and hepatic worsening, characterized by elevated transaminases, prompting the introduction of liposomal amphotericin B (3 mg/kg daily) and temporary suspension of itraconazole. Liposomal amphotericin B was the formulation of choice due to its lower nephrotoxicity profile and availability at the hospital pharmacy. Authorization for outpatient administration of liposomal amphotericin B, either at a dialysis clinic or through home care, was denied by the health insurance provider. Consequently, she completed a full year of inpatient therapy. According to the Clinical Practice Guidelines for the Management of Sporotrichosis (2007 update by the Infectious Diseases Society of America), a total of at least 12 months of therapy is required for disseminated forms of the disease, and chronic suppressive therapy should be considered for immunosuppressed patients [1]. Following this extended hospitalization, she was discharged for outpatient management and to resume treatment for SLE. Laboratory data from the start of treatment, at the time of antifungal medication switch, and at the end of treatment are described in Table 1.

**TABLE 1:** Laboratory markers at diagnosis (June 2024), the moment to switch antifungal medication (August 2024), and end of treatment (June 2025).

	June 2024	August 2024	June 2025	Reference range
Erythrocytes	2.61 10^6^/μL	3.42 10^6^/μL	2.87 10^6^/μL	4.00–5.20·10^6^/μL
Hemoglobin	7.7 g/dL	10.1 g/dL	8.4 g/dL	12.0–16.0 g/dL
Hematocrit	23%.1%	33%	25.6%	36.0%–46.0%
Leukogram	7.040/μL	7.590/μL	8.680/μL	4.500–11.000/μL
C‐reactive protein	31.85 mg/dL	1.43 mg/dL	3.76 mg/dL	< 0.30 mg/dL
Pyruvic transaminase: GPT (alanine aminotransferase)	11 U/L	64 U/L	39 U/L	6–45 U/L
Oxalacetic transaminase: GOT (aspartate aminotransferase)	11 U/L	145 U/L	60 U/L	15–37 U/L
Alkaline phosphatase	262 U/L	509 U/L	1000 U/L	46–116 U/L
Gamma‐glutamyl transferase	276 U/L	705 U/L	659 U/L	5–55 U/L
Urea	174 mg/dL	53 mg/dL	181 mg/dL	15–50 mg/dL
Creatinine	9.22 mg/dL	2.74 mg/dL	7.30 mg/dL	0.50–1.10 mg/dL
Sodium	141 mmol/L	141 mmol/L	136 mmol/L	136–145 mmol/L
Potassium	4.1 mmol/L	3.8 mmol/L	4.2 mmol/L	3.5–5.1 mmol/L

## 3. Discussion

Sporotrichosis is a neglected disease and is considered the most common subcutaneous mycosis in Latin America [2]. This case report describes an unusual presentation of sporotrichosis in a young immunocompromised Brazilian woman. Its etiological agent is *Sporothrix* spp., a genus that includes 53 species, some of which are particularly significant in public health: *S. brasiliensis*, *S. schenckii*, and *S. globosa* [3]. This fungus is typically found in the environment, especially in areas related to gardening, due to its presence in soil, plants, and decaying wood. Over the years, sapronotic transmission has been the most common form of infection; however, zoonotic transmission has been increasingly frequent, particularly with *S. brasiliensis*, where transmission primarily occurs through contact with the skin lesions of infected cats, via bites and scratches. On the other hand, *S. schenckii* is transmitted through fungal inoculation, typically via contaminated plants [6]. This increase in zoonotic transmission by the spread of *S. brasiliensis*, a species known for its high virulence, greater adaptation to the Brazilian climate, and increased thermotolerance [7]. Sporotrichosis has a similar prevalence between sexes, with no preference for age or race [6].

As described in the case report, the initial culture of the hematoma aspirate was negative, a finding that may be attributed to the low fungal load in necrotic or bloody material, reinforcing the importance of tissue biopsy for definitive diagnosis in suspected sporotrichosis [4]. Furthermore, the patient did not initially report contact with cats, and this information was not elicited by the assisting medical team. Only later, during a more comprehensive anamnesis, was the association with feline exposure established. A thorough anamnesis is particularly critical in immunocompromised patients. It is important to emphasize that a detailed investigation of exposures, including animals, may provide essential clues for diagnosing fungal infections in this population [8].

According to the 2022 update from the Brazilian Society of Dermatology, human sporotrichosis is classified clinically as follows: Cutaneous (lymphocutaneous, fixed cutaneous, and multiple inoculation), Mucosal (ocular, nasal, and others), Osteoarticular, Systemic (cutaneous/mucosal, pulmonary, neurological, and others), Immunoreactive, and localized mixed forms [7]. In the present case, the final subtype diagnosis was disseminated cutaneous sporotrichosis, which is one of the more aggressive clinical forms of the disease, more frequently observed in immunocompromised patients. Predisposing conditions include HIV/AIDS, diabetes mellitus, chronic alcoholism, and states of induced immunosuppression, such as in transplant patients [9].

The relationship between sporotrichosis and SLE deserves special attention, as the patient in this report has the disease. SLE is an autoimmune disorder of still incompletely understood etiology, characterized by a chronic multisystem inflammatory pattern. It predominantly affects women, Black individuals, and Asian populations, with higher prevalence between the ages of 20 and 45 [10]. In immunocompetent individuals, infection caused by *Sporothrix* spp. is usually localized, with progression limited by the cellular immune response, particularly through the activation of macrophages and T lymphocytes. However, in immunocompromised patients, there is a reduced efficiency of the inflammatory and phagocytic responses, favoring the spread of the disease. The deficiency in the production of proinflammatory cytokines, such as IFN‐γ and TNF‐α, impairs macrophage activation, facilitating the invasion of deep tissues and systemic organs [11].

One of the main factors contributing to morbidity and mortality in these lupus patients is the increased risk of infections due to significant immunological compromise, characterized by lymphopenia, reduced CD4+ T lymphocytes, neutropenia, and complement system dysfunction [9]. It is estimated that about 50% of the SLE patients will develop at least one severe infectious episode during their lifetime, with 25% of deaths in these individuals being related to infections [10]. Furthermore, in immunocompromised patients, the response to itraconazole may be reduced, requiring prolonged therapeutic regimens or the need for liposomal amphotericin B for infection control [8].

It is evident that sporotrichosis continues to rise, and several factors contribute to the difficulties in managing and treating the disease, as already described. Five main factors stand out: the lack of a public health program for disease control, the absence of a targeted and low‐cost (or free) medication for both humans and animals, the shortage of diagnostic centers, the lack of awareness among the population about the disease, and the difficulty in providing complete treatment for felines [8]. One of the main obstacles in controlling sporotrichosis is the lack of knowledge about its existence, epidemiology, and clinical manifestations, which can even prevent accurate diagnosis [8]. It is emphasized that fungal diseases should always be suspected in immunocompromised patients, especially lupus patients [10].

## 4. Conclusion

The present case illustrates this dangerous intersection, where the reduced efficiency of the cellular immune response facilitates fungal dissemination and may diminish the efficacy of standard treatments. As infections remain a leading cause of morbidity and mortality in SLE patients, this report underscores the critical need for heightened clinical suspicion of fungal diseases in this population.

## Funding

The authors disclosed receipt of the following financial support for the research, authorship, and publication of this article.

## Consent

Authorization for publication of the case study was obtained from the patient.

## Conflicts of Interest

The authors declare no conflicts of interest.

## Data Availability

Medical record data used to support the findings of this case report are restricted to protect patient privacy. Appropriate, specific medical record data have been included verbatim within the article. This case report also presents a discussion of sporotrichosis in a patient with lupus erythematosus. The data used in the discussion were obtained from peer‐reviewed journals and previously published case reports. Appropriate citations and references are included within the article.
